# Pigmentary retinopathy and nodular granuloma associated with acute retinal necrosis from varicella zoster virus and human herpes virus type 6: Case report

**DOI:** 10.1097/MD.0000000000033958

**Published:** 2023-06-30

**Authors:** Narumon Keorochana, Budsarat Suleesathira, Sritatath Vongkulsiri

**Affiliations:** a Department of Ophthalmology, Phramongkutklao Hospital, Phramongkutklao College of Medicine, Bangkok, Thailand.

**Keywords:** acute retinal necrosis, case report, human herpesvirus type 6, nodular granuloma, pigmentary retinopathy, retinitis, varicella zoster virus

## Abstract

Acute retinal necrosis (ARN) caused by human herpes virus type 6 (HHV-6) is uncommon. We described a case of consecutive bilateral ARN, which was found to be a coinfection of varicella zoster virus (VZV) and HHV-6 in a 50-year-old woman, not well responded with systemic acyclovir. We showed the atypical findings with corresponding fundus and optical coherence tomography imaging.

**Patient concerns:**

She presented with anterior segment inflammation with peripheral retinitis and vasculitis in the left eye with disease progression despite of initial antiviral treatment, end up with retinal detachment. The right eye, subsequently, developed focal retinitis.

**Diagnosis:**

ARN was diagnosed by clinical fundus picture, confirmed by polymerase chain reaction (PCR).

**Interventions:**

Initially, she was treated with intravenous acyclovir and intravitreal ganciclovir for left eye. Retinal necrosis progressed, followed by retinal detachment. Pars plana vitrectomy with silicone oil was performed. The right eye, subsequently, developed focal retinitis. Medication was switched to intravenous ganciclovir and then oral valganciclovir.

**Outcomes:**

Retinitis was resolved, generalized hyperpigmentation appeared as a salt-and-pepper appearance in the right eye. The left eye presented preretinal deposits on silicone-retina interphase along retinal vessels. Spectral-domain optical coherence tomography (SD-OCT) showed multiple hyperreflective nodules on retinal surface.

**Lessons:**

ARN from coinfection of VZV and HHV-6 is rare. Preretinal granulomas and generalized hyperpigmentation could be one of the HHV-6 features. HHV-6 should be in the differential diagnosis for ARN. It responds well to systemic ganciclovir.

## 1. Introduction

Acute retinal necrosis (ARN) is an infectious disease of the retina, first reported in 1971 by Urayama from Japan.^[1]^ It presents full-thickness retinal necrosis at the peripheral retina together with prominent vitritis and occlusive vasculopathy.^[2]^ The incidence of ARN was 0.63 cases per 1 million reported in the UK,^[3]^ while 1.9% of patients with uveitis was diagnosed with ARN in a tertiary care hospital in Thailand.^[4]^ Even though ARN is not a common disease, it can cause high morbidity and lead to blindness especially when the diagnosis and treatment are delayed. Varicella zoster virus (VZV) and herpes simplex virus (HSV) type1, 2 are the most common causes of the disease, however, some other viruses also constitute causative agents, such as Cytomegalovirus (CMV) and Epstein Barr virus (EBV).^[2,5]^ We reported atypical clinical findings and refractory treatment response in ARN from coinfection of VZV and human herpes virus 6 (HHV-6).

## 2. Case report

In December 2020, a healthy 50-year-old Thai woman presented at Phramongkutklao Hospital with decreased vision and floaters in the left eye for 1 week. The patient had no underlying disease and relevant social and surgical history. Nevertheless, she reported a history of shingles around the neck 10 years ago.

**Figure 1.**
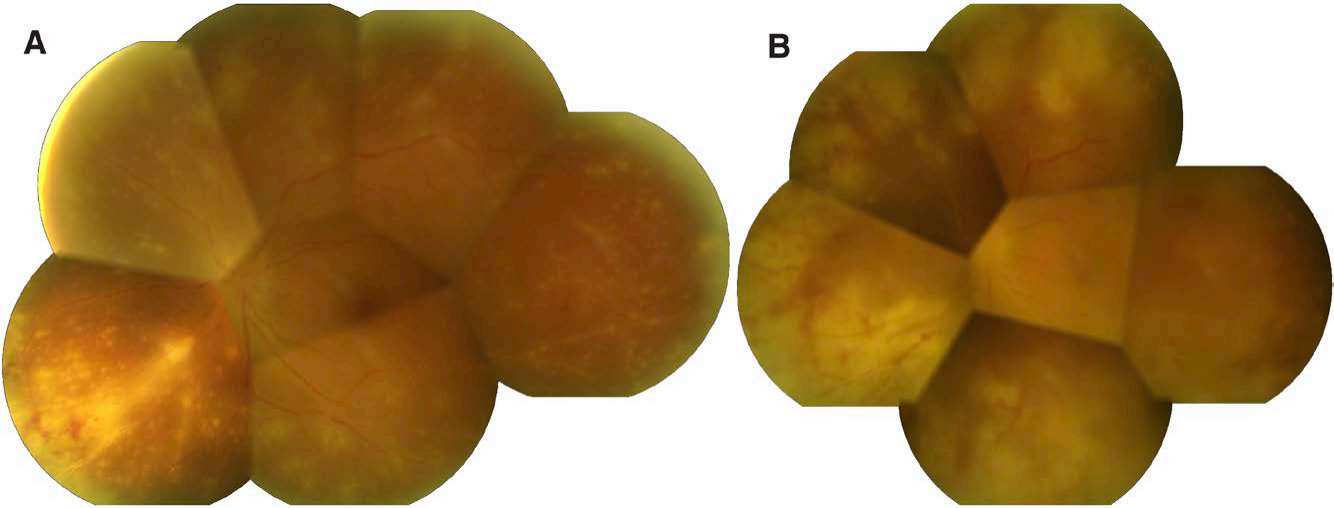
Montage fundus photography in the left eye showed (A) optic disc swelling, diffuse multiple whitish retinitis at peripheral retina with arteriolitis and phlebitis. (B) Peripheral retinitis with vasculitis progressed even antiviral treatment for 5 d.

Initial examination showed 20/100 visual acuity in the left eye using pinhole examination. Intraocular pressure was 11 mm Hg, and relative afferent pupillary defect was present in the left eye. The anterior segment revealed injected conjunctiva with corneal descemet fold, 3 + anterior chamber cells and multiple whitish mutton fat granulomatous keratic precipitates at Art triangle. Grade 1 vitreous haze with 2 + cells in the anterior vitreous, hyperemic optic disc with blurred margin, diffuse multiple whitish retinitis accompanied with arteriolitis and phlebitis at the peripheral retina were presented (Fig. 1A). The right eye was unremarkable with a visual acuity of 20/25 using pinhole examination. The patient received a diagnosis of ARN in the left eye and was admitted starting treatment with intravenous acyclovir 10 mg/kg/dose every 8 hours and supplemented with intravitreal 2 mg/0.1 mL ganciclovir injection in the left eye. The diagnosis was confirmed using aqueous polymerase chain reaction (PCR) in which VZV and HHV type 6 were detected. After starting treatment with acyclovir for 2 days, the patient received oral prednisolone 1 mg/kg/day to control the inflammation. Initial infectious workups were negative including anti-HIV, treponema pallidum hemagglutination and venereal disease research laboratory test. Chest X-ray was normal.

**Figure 2.**
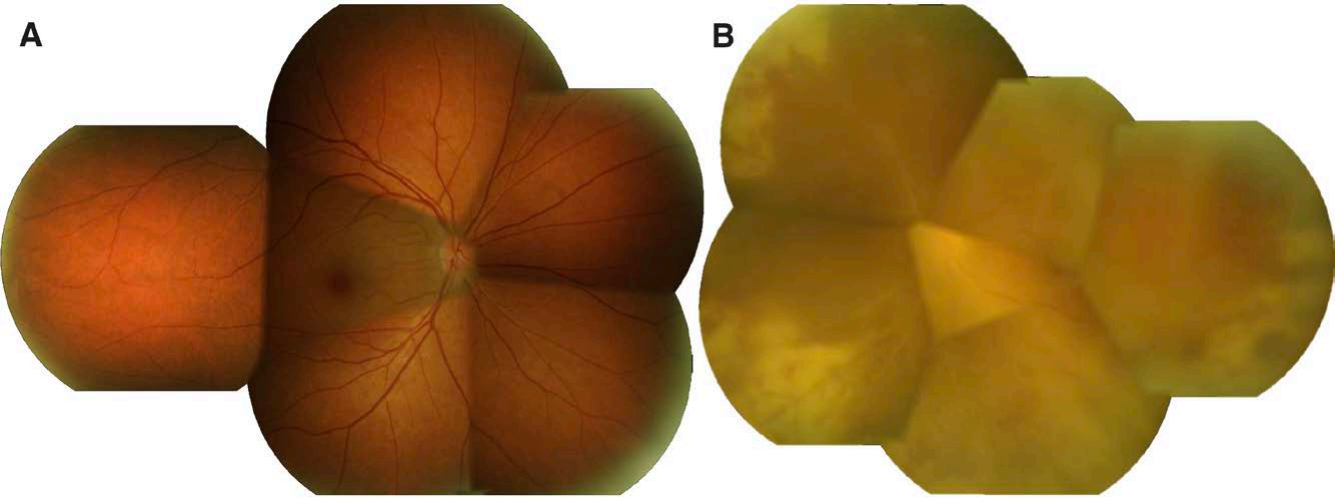
Montage fundus photography after intravenous acyclovir treatment for 17 d demonstrated (A) normal in right eye. (B) Left eye showed progression of severe vitritis, confluent circumferential of retinitis and inferior rhegmatogenous retinal detachment was detected in peripheral retina.

The confluence of retinal infiltration was observed. Retinitis and vasculitis had progressed to midperiphery after administering acyclovir and steroid for 5 days (Fig. 1B). The acyclovir was increased to 12 mg/kg/dose every 8 hours and prednisolone was discontinued. The clinical condition did not improve even though acyclovir had been administered for 17 days. Progressively confluent area of full-thickness retinal necrosis with inferior rhegmatogenous retinal detachment finally occurred (Fig. 2). The acyclovir increased to 15 mg/kg/dose and pars plana vitrectomy with silicone oil was performed. Vitreous samples were sent for bacterial and fungal culture, cytology and PCR to determine the Herpesviridae family. VZV and HHV type 6 were detected, which were consistent with prior aqueous PCR results. Other work-up results were negative.

After 3 weeks of intravenous acyclovir together with pars plana vitrectomy with silicone oil in the left eye, the retinitis did not subside. Furthermore, new retinitis foci developed at the inferonasal area of the far-peripheral retina in the right eye. We decided to switch the antiviral medication from intravenous acyclovir to ganciclovir 5 mg/kg/dose every 12 hours for 2 weeks followed by valganciclovir 450mg twice daily as a maintenance therapy for 4 months.

**Figure 3.**
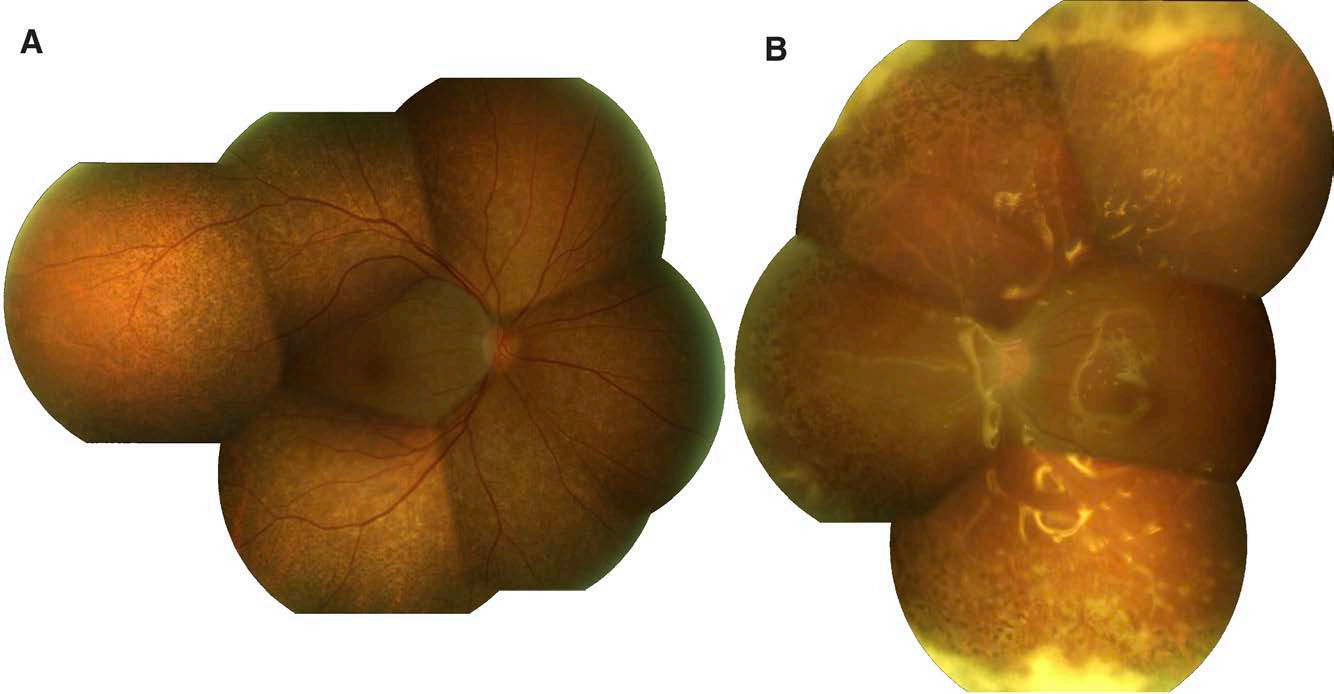
Montage fundus photography after switching treatment to intravenous ganciclovir for 3 wk revealed (A) resolution of retinitis and development of generalized hyperpigmentation throughout the retina in right eye. (B) Left fundus showed attached retina with improvement of retinitis, turning to peripheral fibrotic scar. Multiple focal whitish perivascular deposit along both artery and vein in the posterior pole were noted especially along inferior vasculature.

**Figure 4.**
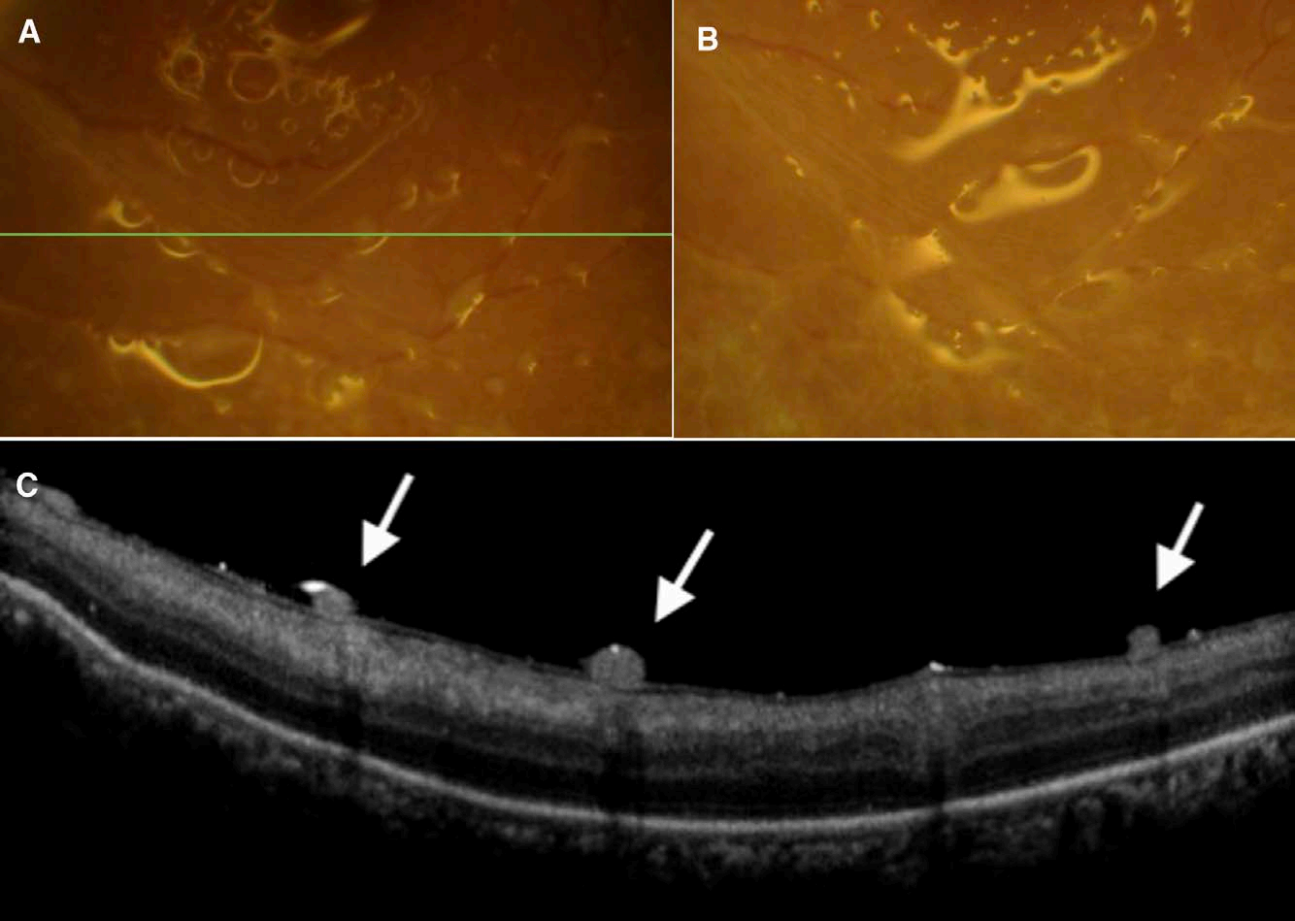
(A) Fundus photography of right eye showed generalized hyperpigmentation as salt-and-pepper appearance with corresponding (B) hyper- and hypo-autofluorescence in fundus autofluorescence. (C) SD-OCT at foveal center showed preserving external limiting membrane and ellipsoid zone with central hyperreflective lesion at ellipsoid and interdigitation zone. (D) Perifoveal SD-OCT revealed peaked hyperreflective foci of focal thickening retinal pigment epithelial layer. SD-OCT = spectral-domain optical coherence tomography.

**Figure 5.**
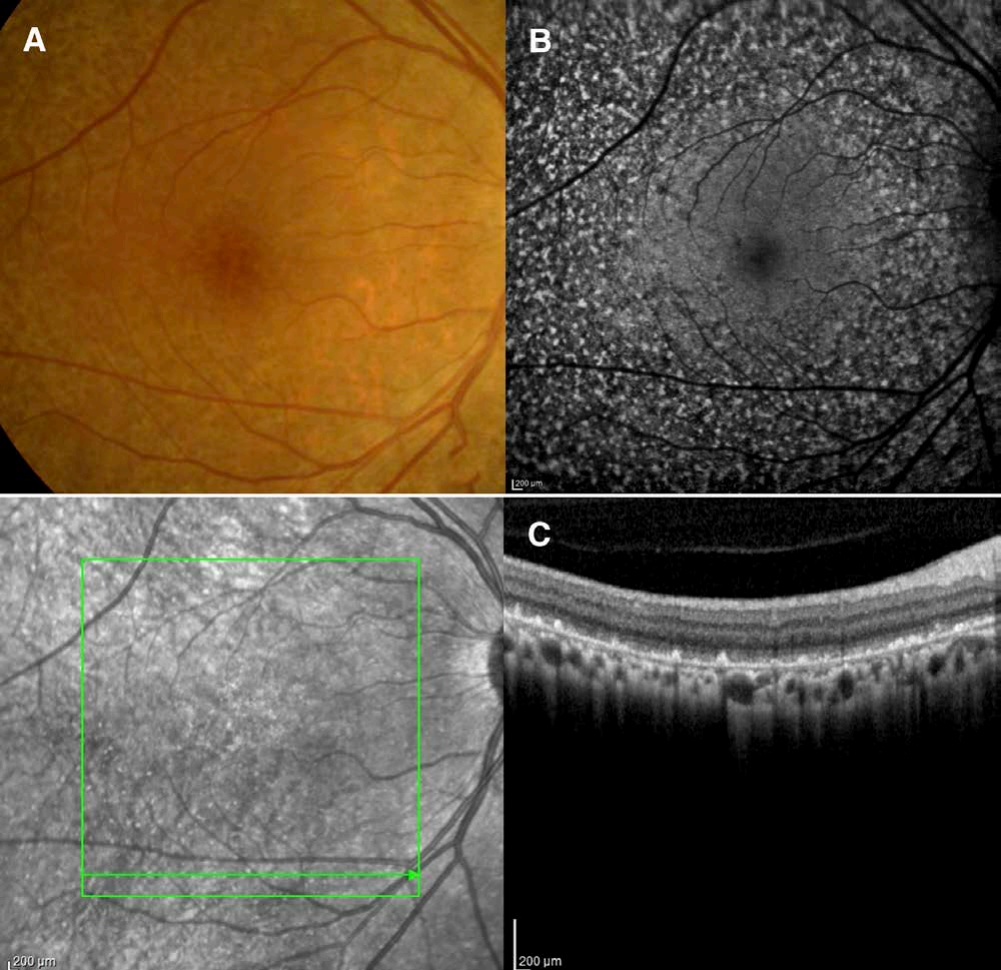
Fundus photography focused on inferior vascular arcade demonstrated (A) multiple round whitish granuloma-liked deposits along blood vessels. (B) The lesions gradually resolved and hypopigmented along the attenuated blood vessels with time. (C) SD-OCT through the deposits (green line in A) showed multiple homogeneous hyperreflective nodules abutting the retinal surface nearby the blood vessels. Vessels showed as vertical hyperreflective oval lesions in nerve fiber layer with shadowing. (D) Foveal OCT also revealed hyperreflective nodules in silicone-retina interface with disorganized hyperreflective retinal layer of foveal center. SD-OCT = spectral-domain optical coherence tomography.

Three weeks after intravenous ganciclovir injection, retinitis was resolved in the right eye and turned to generalized hyperpigmentation, salt-and-pepper appearance, throughout the retina (Figs. 3A and 4A). Autofluorescence imaging showed hyper- and hypo-autofluorescence corresponding to color fundus photography (Fig. 4B). Spectral-domain optical coherence tomography (SD-OCT) at the points of hyperpigmentation lesions revealed thickening hyperreflective foci of retinal pigment epithelium (Fig. 4C and D). Multiple round-shaped whitish granulomas deposited along both retinal arteries and veins and on the retinal surface around the posterior pole were detected in the left eye (Figs. 3B and 5A). SD-OCT showed multiple hyperreflective nodules on the retinal surface near the blood vessels showing as vertical hyperreflective oval lesions in the nerve fiber layer with shadowing (Fig. 5C). After continuing oral valganciclovir for 4 months, the granulomas along the vessels decreased in size but whitish preretinal granulomas appeared on the macular area in the left eye. However, the best corrected visual acuity, 20/20 in the right eye and finger count of 3 feet in the left eye, were unchanged. We decided to stop oral valganciclovir and closely follow up. After discontinuing oral valganciclovir for 2 months, the granulomas in the posterior pole decreased in size and number with time.

## 3. Discussion

We presented a case diagnosed with ARN with complete diagnostic criteria from the American Uveitis Society^[6]^ including one or more foci of retinal necrosis with discrete borders, located in the peripheral retina, rapid progression in the absence of antiviral therapy, circumferential spreading, occlusive vasculopathy with arterial involvement, a prominent inflammatory reaction in the vitreous and anterior chamber and optic neuropathy. The aqueous and vitreous PCR confirmed positive for VZV and HHV-6 which were undetected in normal populations.^[7]^

Many studies reported the Herpesviridae family such as VZV, HSV-1, HSV-2, CMV, and EBV^[8–11]^ constituted a causative agent in ARN; however, little literature reported HHV-6 infection.^[12–16]^ HHV-6 was the causative agent of exanthem subitum which was separated in 2 distinct viruses as HHV-6A and HHV-6B. HHV-6B was detected in intraocular inflammation more than HHV-6A which was usually detected in the central nervous system.^[13]^ Our case clinically presented as ARN with a coinfection of VZV and HHV-6, without subtype differentiation due to laboratory limitations and was detected by PCR as the causative agent.

For optic neuritis and retinal vasculitis from HHV-6, in which inflammation is predominately caused by an immune response, systemic steroid alone was effective for treatment.^[14]^ Nevertheless, in case of ARN, antiviral therapy was the mainstay of therapy. The patient responded well to acyclovir^[15]^ while ganciclovir, foscarnet, cidofovir and long term valganciclovir were effective treatments in many case reports.^[16–19]^ Hill JA^[20]^ reported that high dose foscarnet 90 mg/kg every 12 hours and ganciclovir 5 mg/kg every 12 hours for induction were both similarly effective to treat HHV-6 base on clinical,^[21]^ in vitro activity^[22]^ and evidence of in vivo effectiveness.^[23]^ As aforementioned, they might be the reason intravenous acyclovir combined with intravitreal ganciclovir and systemic steroid was ineffective at the beginning of the treatment in our patient. After switching intravenous acyclovir to intravenous ganciclovir, the clinical picture improved. However, long term valganciclovir was needed. In summary, our case received intravenous acyclovir for 3 weeks, then switched to intravenous ganciclovir for 2 weeks followed by oral valganciclovir for 4 months.

While receiving intravenous ganciclovir, the right fundus developed generalized hyperpigmentation which had never been reported in viral retinitis from VZV, HSV, EBV, or CMV. Generalized hyperpigmentation in our case appeared like the salt-and-pepper fundus picture of rubella infection which is also a viral infection. Even though unilateral pigmentary retinopathy is rare, it has been reported in acquired rubella infection among adult.^[24]^ We postulated these clinical features were due to HHV-6. After rhegmatogenous retinal detachment was treated by vitrectomy with silicone oil in the left eye, the multiple preretinal nodular deposits on retinal surface along the arteries and veins in posterior pole were noted. The homogeneous hyperreflective deposits on the retinal surface nearby the blood vessels were seen in SD-OCT. These were similar to amorphous deposits^[25]^ and preretinal granulomas^[26–29]^ from toxoplasma retinochoroiditis. These nodules differed clinically from kyrieleis arteritis but had the same fluorescein angiography characteristics.^[26]^ However, no further investigation was conducted to confirm toxoplasma infection in our case. On another thought, the nodular deposits could be the inflammatory reaction to silicone oil itself which has been reported as preretinal hyperreflective organized coarse material in silicone-retina interphase.^[30]^ It had been observed in 14% of the patient undergoing retinal detachment surgery with silicone oil, similar to our scenario and disappeared after silicone oil removal.^[30]^ In our case, it seemed to move posteriorly on the retinal surface from the perimacular area, initially along the vascular arcade, to the macular area. The nature of deposits appeared like movable nodules in the silicone-retina interphase which were stuck on the step of the prominent retinal vasculature and gravitational move to the macula. Finally, gradual resolution of the lesions was observed.

## 4. Conclusion

ARN from coinfection of VZV and HHV-6 is rare and difficult to treat. The preretinal granulomas and generalized hyperpigmentation after retinitis was resolved must be in the differential diagnosis of ARN from HHV-6. Intraocular fluid PCR is very useful for diagnosis. Furthermore, it responds well to intravenous ganciclovir treatment followed by oral valganciclovir.

## Author contributions

**Conceptualization:** Narumon Keorochana.

**Data curation:** Narumon Keorochana, Budsarat Suleesathira.

**Investigation:** Sritatath Vongkulsiri.

**Project administration:** Narumon Keorochana, Sritatath Vongkulsiri.

**Supervision:** Sritatath Vongkulsiri.

**Writing – original draft:** Narumon Keorochana, Budsarat Suleesathira.

**Writing – review & editing:** Sritatath Vongkulsiri.

## References

[1] Urayama A Yamada N Sasaki T. Unilateral acute uveitis with retinal periarteritis and detachment. Jpn J Clin Ophthalmol. 1971;25:607–19.

[2] Cochrane TF Silvestri G McDowell C. Acute retinal necrosis in the United Kingdom: results of a prospective surveillance study. Eye (Lond). 2012;26:370–7; quiz 378. 22281865 10.1038/eye.2011.338 PMC3298997

[3] Muthiah MN Michaelides M Child CS. Acute retinal necrosis: a national population-based study to assess the incidence, methods of diagnosis, treatment strategies and outcomes in the UK. Br J Ophthalmol. 2007;91:1452–5. 17504853 10.1136/bjo.2007.114884 PMC2095441

[4] Keorochana N. Pattern and outcome of uveitis in a tertiary military hospital in Thailand. Ocul Immunol Inflamm. 2020;28:424–32. 31009267 10.1080/09273948.2019.1589527

[5] Goldstein DA Pyatetsky D. Necrotizing Herpetic Retinopathies. Focal Points: Clinical Modules for Ophthalmologists. San Francisco, CA: American Academy of Ophthalmology; 2008. module 10.

[6] Holland GN. Executive committee of the American uveitis society. Standard diagnostic criteria for the acute retinal necrosis syndrome. Am J Ophthalmol. 1994;117:663–7. 8172275 10.1016/s0002-9394(14)70075-3

[7] Keorochana N Intaraprasong W Choontanom R. Herpesviridae prevalence in aqueous humor using PCR. Clin Ophthalmol. 2018;12:1707–11. 30233134 10.2147/OPTH.S174694 PMC6135072

[8] Miserocchi E Iuliano L Fogliato G. Bilateral acute retinal necrosis: clinical features and outcomes in a multicenter study. Ocul Immunol Inflamm. 2018;27:1090–8. 30059636 10.1080/09273948.2018.1501494

[9] Hillenkamp J Nolle B Bruns C. Acute retinal necrosis: clinical features, early vitrectomy, and outcomes. Ophthalmology. 2009;116:1971–5.e2. 19592111 10.1016/j.ophtha.2009.03.029

[10] Meghpara B Sulkowski G Kesen MR. Long-term follow-up of acute retinal necrosis. Retina. 2010;30:795–800. 20057342 10.1097/IAE.0b013e3181c7013c

[11] Vann VR Atherton SS. Neural spread of herpes simplex virus after anterior chamber inoculation. Invest Ophthalmol Vis Sci. 1991;32:2462–72. 1714427

[12] Cohen J Fahle G Kemp M. Human herpesvirus 6-A, 6-B, and 7 in vitreous fluid samples. J Med Virol. 2010;82:996–9. 20419813 10.1002/jmv.21751 PMC2938775

[13] Sugita S Shimizu N Watanabe K. Virological analysis in patients with human herpes virus 6-associated ocular inflammatory disorders. Invest Ophthalmol Vis Sci. 2012;53:4692–8. 22700707 10.1167/iovs.12-10095

[14] Ogata N Koike N Yoshikawa T. Human herpesvirus 6-associated uveitis with optic neuritis diagnosed by multiplex PCR. Jpn J Ophthalmol. 2011;55:502–5. 21814813 10.1007/s10384-011-0069-4

[15] Papageorgiou E Ch’ng S Kulkarni A. Fourth cranial nerve palsy and bilateral acute retinal necrosis following human herpesvirus 6 infection of the central nervous system. Ocul Immunol Inflamm. 2014;22:228–32. 24328436 10.3109/09273948.2013.856533

[16] Maslin J Bigaillon C Froussard F. Acute bilateral uveitis associated with an active human herpesvirus-6 infection. J Infect. 2007;54:e237–40. 17303245 10.1016/j.jinf.2006.12.012

[17] Oberacher-Velten IM Jonas JB Jünemann A. Bilateral optic neuropathy and unilateral tonic pupil associated with acute human herpesvirus 6 infection: a case report. Graefes Arch Clin Exp Ophthalmol. 2005;243:175–7. 15742213 10.1007/s00417-004-0986-8

[18] Moschettini D Franceschini R Vaccaro NM. Human herpesvirus-6B active infection associated with relapsing bilateral anterior optic neuritis. J Clin Virol. 2006;37:244–7. 17005444 10.1016/j.jcv.2006.08.018

[19] Méchaï F Boutolleau D Manceron V. Human herpesvirus 6-associated retrobulbar optic neuritis in an HIV-infected patient: response to anti-herpesvirus therapy and long-term outcome. J Med Virol. 2007;79:931–4. 17516535 10.1002/jmv.20833

[20] Hill JA. Human herpesvirus 6 in transplant recipients: an update on diagnostic and treatment strategies. Curr Opin Infect Dis. 2019;32:584–90. 31567413 10.1097/QCO.0000000000000592 PMC7141773

[21] Tomblyn M Chiller T Einsele H. National Marrow Donor program; European Blood and MarrowTransplant Group; American Society of Blood and Marrow Transplantation; Canadian Blood and Marrow Transplant Group; Infectious Diseases Society of America; Society for Healthcare Epidemiology of America; Association of Medical Microbiology and Infectious Disease Canada; Centers for Disease Control and Prevention. Guidelines for preventing infectious complications among hematopoietic cell transplantation recipients: a global perspective. Biol Blood Marrow Trans. 2009;15:1143–238. 10.1016/j.bbmt.2009.06.019 PMC3103296 19747629

[22] Chemaly RF Hill JA Voigt S. In vitro comparison of currently available and investigational antiviral agents against pathogenic human double-stranded DNA viruses: a systematic literature review. Antiviral Res. 2019;163:50–8. 30677427 10.1016/j.antiviral.2019.01.008

[23] Zerr DM Gupta D Huang ML. Effect of antivirals on human herpesvirus 6 replication in hematopoietic stem cell transplant recipients. Clin Infect Dis. 2002;34:309–17. 11774077 10.1086/338044

[24] Damasceno N Damasceno E Souza E. Acquired unilateral rubella retinopathy in adult. Clin Ophthalmol. 2010;5:3–4. 21311649 10.2147/OPTH.S15273 PMC3032996

[25] Guagnini AP De Potter P Levecq L. Atypical spherical deposition on vitreoretinal interface associated with toxoplasmic chorioretinitis. Graefes Arch Clin Exp Ophthalmol. 2007;245:158–60. 16612633 10.1007/s00417-006-0330-6

[26] Oliver GF Ferreira LB Vieira BR. Posterior segment findings by spectral-domain optical coherence tomography and clinical associations in active toxoplasmic retinochoroiditis. Sci Rep. 2022;12:1156. 35064148 10.1038/s41598-022-05070-9 PMC8782858

[27] Tolou C Salmon L Mahieu L. Spectral-domain optical coherence tomography appearance of preretinal granulomas in toxoplasma posterior uveitis with arterial occlusion. J Fr Ophtalmol. 2015;38:889–91. 26358432 10.1016/j.jfo.2015.02.006

[28] Goldenberg D Goldstein M Loewenstein A. Vitreal, retinal, and choroidal findings in active and scarred toxoplasmosis lesions: a prospective study by spectral-domain optical coherence tomography. Graefes Arch Clin Exp Ophthalmol. 2013;251:2037–45. 23568271 10.1007/s00417-013-2334-3

[29] Invernizzi A Agarwal AK Ravera V. Comparing optical coherence tomography findings in different aetiologies of infectious necrotising retinitis. Br J Ophthalmol. 2018;102:433–7. 28765144 10.1136/bjophthalmol-2017-310210

[30] Trivizki O Zur D Goldenberg D. A novel finding of hyperreflective material in the silicone-retina interface: an optical coherence tomographic and histopathological study. Retina. 2020;40:2055–60. 31688670 10.1097/IAE.0000000000002691

